# Association of coagulase-negative staphylococci with orthopedic infections detected by in-house multiplex real-time PCR

**DOI:** 10.3389/fmicb.2024.1400096

**Published:** 2024-06-07

**Authors:** Ying Wang, Chao Liu, Wenbo Xia, Yanxiang Cui, Linhong Yu, Dan Zhao, Xiaoxuan Guan, Yingdi Wang, Yani Wang, Yisong Li, Jianqiang Hu, Jie Liu

**Affiliations:** ^1^School of Public Health, Qingdao University, Qingdao, Shandong, China; ^2^Department of Orthopedics, Qingdao Huangdao Traditional Chinese Medicine Hospital, Qingdao, Shandong, China; ^3^Department of Clinical Laboratory, Qingdao Huangdao Traditional Chinese Medicine Hospital, Qingdao, Shandong, China; ^4^Qingdao Medical College, Qingdao University, Qingdao, Shandong, China

**Keywords:** coagulase-negative staphylococci, orthopedics, infections, real-time polymerase chain reaction, multiplex, etiology

## Abstract

**Introduction:**

Clinical significance of coagulase-negative staphylococci (CoNS) has been gradually acknowledged in both healthcare and clinical research, but approaches for their precise discrimination at the species level remain scarce. The current study aimed to evaluate the association of CoNS with orthopedic infections, where accurate and prompt identification of etiology is crucial for appropriate diagnosis and treatment decision-making.

**Methods:**

A 16S rRNA-based quantitative PCR (qPCR) assay was developed for the detection of *Staphylococcus* genus and two panels of 3-plex qPCR assays for further differentiation of six CoNS species with remarkable clinical significance, including *S. epidermidis*, *S. haemolyticus*, *S. simulans*, *S. hominis*, *S. capitis*, and *S. caprae*. All the assays exhibited excellent analytical performance. ΔCq (quantification cycle) between 16S rRNA and CoNS species-specific targets was established to determine the primary CoNS. These methods were applied to detect CoNS in wound samples from orthopedic patients with and without infection.

**Results and discussion:**

Overall, CoNS were detected in 17.8% (21/118) of patients with clinically suspected infection and in 9.8% (12/123) of patients without any infection symptom (*p* < 0.05). Moreover, the association with infection was found to be bacterial quantity dependent. *S. epidermidis* was identified as the predominant species, followed by *S. simulans*, *S. haemolyticus*, and *S. hominis*. Male sex, open injury, trauma, and lower extremity were determined as risk factors for CoNS infections. CoNS-positive patients had significantly longer hospitalization duration (20 days (15, 33) versus 13 days (7, 22) for *Staphylococcus*-negative patients, *p* = 0.003), which could be a considerable burden for healthcare and individual patients. Considering the complex characteristics and devastating consequences of orthopedic infections, further expanding the detection scope for CoNS may be pursued to better understand the etiology of orthopedic infections and to improve therapeutic strategies.

## 1 Introduction

Orthopedic infections, including foreign body-related infections, are notoriously difficult to treat, with a high risk of leaving patients disabled or even dead (Tande and Patel, 2014; Glaudemans et al., 2019; Moriarty et al., 2022). Therefore, precise and timely diagnosis is crucial for prompt treatment decision. The causative agents for orthopedic infections have been often found to be opportunistic pathogens that colonize on the skin (Méric et al., 2018; Garcia et al., 2019), where staphylococci are one of the most commonly identified bacteria.

Among staphylococci, *Staphylococcus aureus* has been attracting tremendous attention worldwide, as a leading cause of infectious diseases in both human and animal (Turner et al., 2019). In contrast, the pathogenic propensity of another group of *Staphylococcus* genus, namely coagulase-negative staphylococci (CoNS), has been largely ignored until the end of 1980s. Even today, a limited number of studies have been dedicated to reveal the underlying molecular mechanism of CoNS infections (Becker et al., 2014; Heilmann et al., 2019; Michels et al., 2021). As part of human skin and mucosa microbiota, CoNS were historically recognized as a whole, represented by *S. epidermidis*, being less virulent and pathogenic (Severn and Horswill, 2023). However, more and more scientific evidence indicates there are genetic and functional diversities among different CoNS species, which can play an important role in nosocomial infections. Therapeutically, infections caused by CoNS are challenging to cure mostly because they are routinely overlooked in clinical practice, particularly when *S. aureus* is present. Therefore, transmission and outbreak of CoNS in healthcare facilities or communities most likely remain unidentified. Furthermore, the elaborated spectrum of antibiotic resistance and significant capability of biofilm formation make CoNS even more refractory to clinical treatment. To date, the species with most clinical significance include *S. epidermidis*, *S. haemolyticus*, *S. hominis*, *S. lugdunensis*, and *S. capitis* (Campos-Peña et al., 2014; Heilmann et al., 2019; Becker et al., 2020). *S. epidermidis* is the most common species of CoNS infection as one of the most prevalent causative pathogens of implant-associated infections in the United States (Oliveira et al., 2018). *S. haemolyticus* is considered second most important CoNS responsible for nosocomial infections (Kim et al., 2018). *S. lugdunensis* resembles many key characteristics of *S. aureus*, mostly causing infectious endocarditis (Petti et al., 2008; Argemi et al., 2017; Heilbronner and Foster, 2021). Nowadays, besides *S. epidermidis*, most studies often analyze CoNS altogether, instead of distinguishing different species. Therefore, the less frequently encountered species might have been underestimated in the real world (Becker et al., 2014; Michels et al., 2021). Considered the alarming pathogenic significance of CoNS, accurate species-specific identification approaches are in urgent need to discriminate closely related CoNS to improve their therapeutic management (Kim et al., 2018).

With the development of modern molecular technology, our toolbox for organism identification and differentiation has been largely expanded. For instance, whole genome sequencing (WGS) and bioinformatic analysis drastically deepen our understanding of the genetic traits and evolutionary history of microbes. Compared with traditional culture-based microbiological approaches, molecular techniques, such as polymerase chain reaction (PCR), have been exhibiting marked advantages in diagnostic differentiation of causative organisms (Yang and Rothman, 2004; Schmitz et al., 2022). In clinical practice, as the gold standard of pathogen identification, microbial culture can take up to 14 days, while PCR can be done within a couple of hours (Cazanave et al., 2013). Conventional microbiological procedures can be much more laborious or even inconclusive when it comes to unculturable microorganisms or under the interference of previous antibiotic treatment, and the intrinsic flaws of culture-based methods can also sabotage quantitative assessments (Liu et al., 2015). On the contrary, PCR is more sensitive with higher specificity, which has been practically advanced by fluorescent reporter probe. Multiplex quantitative real-time PCR (qPCR) can simultaneously detect different targets in one single reaction. Moreover, the capability of quantification, even from complex samples, makes qPCR a competent technique for pathogen detection (Espy et al., 2006; Schmitz et al., 2022).

In the current study, we developed one *Staphylococcus* genus-specific 16S rRNA qPCR assay and two TaqMan probe based 3-plex qPCR panels to specifically identify six different CoNS species to explore their association with orthopedic infection and evaluate the risk factors for such infections (Campos-Peña et al., 2014; Kim et al., 2018).

## 2 Materials and methods

### 2.1 Study settings

**Figure 1:**
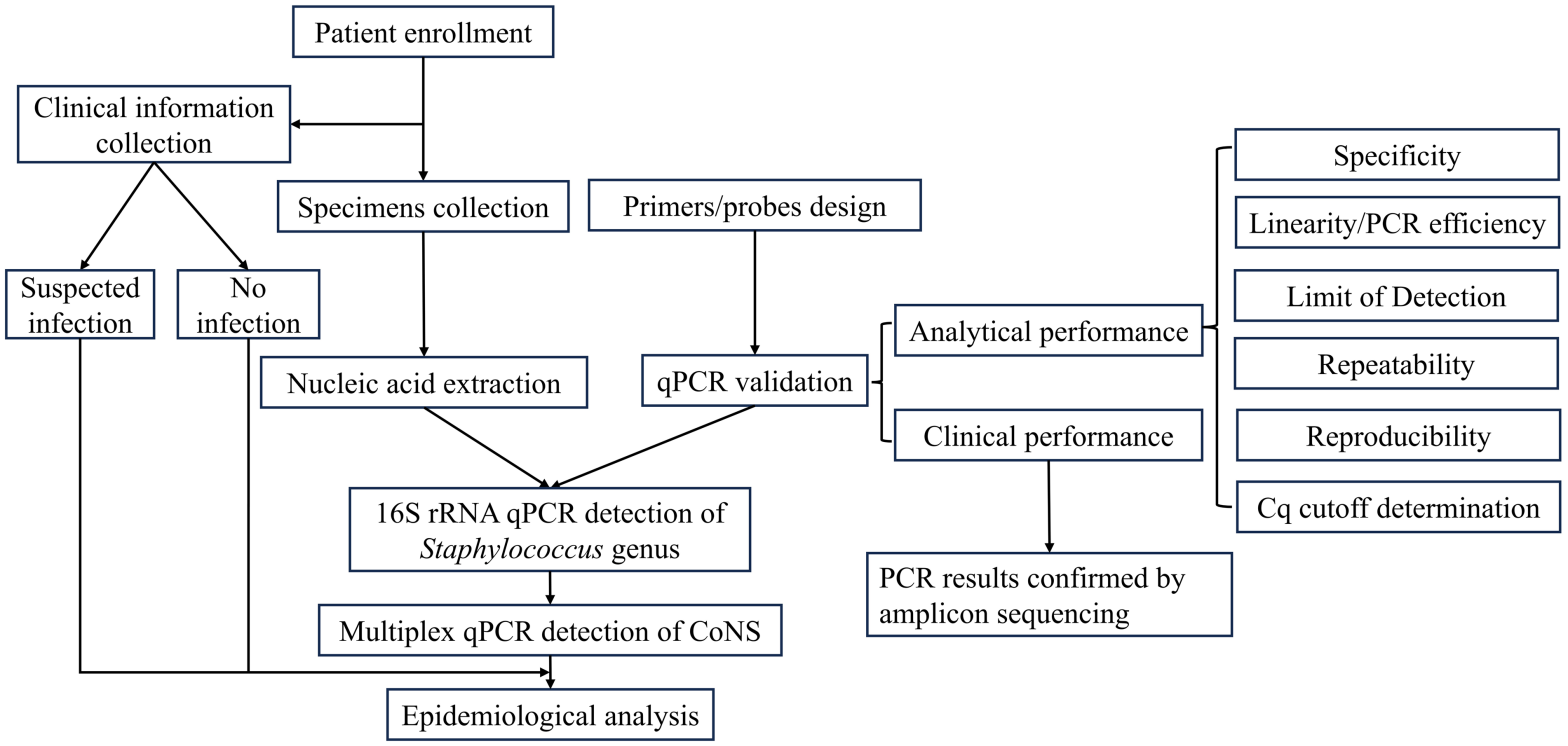
The work flow of the current study.

Inpatients admitted between November 2020 and March 2023 by the department of orthopedics, Qingdao Huangdao Traditional Chinese Medicine Hospital, China were enrolled for the current study. The enrollment criteria included being hospitalized no less than 24 h with integral medical records and having at least one specimen collected for microbiology and qPCR testing. They were grouped based on suspected infection (Group A) or not (Group B) presented upon admission (Table 1). Patients were designated to Group A if ≥1 of the following manifestations matched: open wound with severe contamination; open injury without any hospital visit for more than 24 h; red, swollen, and tender skin associated with ulceration, fistula, sinus tract, or purulent exudate; red and swollen joint with pain and high body temperature; raised serum inflammatory biomarkers, such as white blood cell count (WBC), C-reactive protein (CRP), and erythrocyte sedimentation rate (ESR); abnormal punctate or purulent synovial fluid; abscesses with visible purulent discharge; red, swollen, or tender incision with discharge. Patients of bone tuberculosis or diabetic foot were excluded, because they should be admitted by other departments as soon as diagnosed according to hospital’s administrative policies. All enrolled patients were informed and signed consent documents. Medical charts were reviewed and categorized with EpiData software (epidata.dk, Denmark). This study has been approved by ethics boards of both participant institutes, Qingdao University and Qingdao Huangdao Traditional Chinese Medicine Hospital. All procedures were performed in accordance with the 1964 declaration of Helsinki and later amendments. The entire work flow of the current study is visualized in Figure 1.

**Table 1:** Characteristics of 279 enrolled patients and detection of CoNS species by qPCR.

Characteristics	Group A (*n* = 153)	Group B (n=126)	*P* value
Age (years)			0.151
Median	52	50	
Range	10-94	13-91	
Gender (*n*, %)			0.184
Male	112 (73.20)	83 (65.87)	
Female	41 (26.80)	43 (34.13)	
Hospitalization (days)			0.127
Median	15	13	
Range	1-96	2-85	
Diagnosis (*n*, %)			**<0.001**
Open injury	83 (54.25)*^a^*	31 (24.60)*^b^*	
Closed injury	42 (27.45)*^a^*	88 (69.84)*^b^*	
Postoperation	28 (18.30)*^a^*	7 (5.56)*^b^*	
Cause of injury (*n*, %)			**0.001**
Traffic accident	9 (5.88)	12 (9.52)	
Trauma	83 (54.25)*^a^*	86 (68.26)*^b^*	
Spontaneous disease	31 (20.26)	23 (18.25)	
Cutting	9 (5.88)	2 (1.59)	
Others	21 (13.73)*^a^*	3 (2.38)*^b^*	
Injured site (*n*, %)			0.903
Upper extremity	29 (18.95)	20 (15.87)	
Lower extremity	93 (60.79)	77 (61.11)	
Trunk	5 (3.27)	5 (3.97)	
Whole body	25 (16.34)	24 (19.05)	
Head and face	1 (0.65)	0 (0.00)	
Fracture location			**<0.001**
NO	101 (66.01)*^a^*	45 (35.71)*^b^*	
Upper limb	10 (6.53)	15 (11.90)	
Pelvic limb	24 (15.69)*^a^*	46 (36.51)*^b^*	
Head and face	0 (0.00)*^a^*	5 (3.97)*^b^*	
Trunk	5 (3.27)	6 (4.76)	
Whole body	13 (8.50)	9 (7.15)	

*^a, b^* Differences between groups with statistical significance. Statistical significance was marked in bold.

### 2.2 Collection of clinical samples and identification of clinical isolates

Clinical specimens, including wound secretion, drainage, aspirate, and joint effusion, were collected in duplicate with swabs from participants. One was immediately stored at −80°C until PCR testing, and the other one was streaked out on blood agar or China blue agar plates (Babio, Jinan, China) right upon arrival at the clinical laboratory and incubated at 37°C for 24 h, then single colonies were tested by VITEK® 2 COMPACT automatic microbiology analyzer for identification (bioMérieux, Marcy L’Etoile, France).

### 2.3 Nucleic acid extraction

Clinical isolates were suspended with 100 μL TE buffer (10 mM Tris–HCl and 1 mM EDTA), incubated at 95°C for 30 min and then centrifuged at top speed at 4°C for 5 min. The lysate was used for PCR (Zhu et al., 2006; Liu J. et al., 2012; Martzy et al., 2019). Genomic DNA of *S. epidermidis*, *S. haemolyticus*, *S. simulans*, and *S. hominis* was prepared with bacterial genomic DNA extraction kit (Bioteke Corporation, Beijing, China) following manufacturer’s construction, while target DNA fragments of *S. capitis* and *S. caprae* were synthesized (Tsingke Biological Technology, Beijing, China).

Swab samples were soaked and washed with 200 μL PBS buffer to extract nucleic acid either manually with extraction kit mentioned above or QIAcube HT system (Qiagen, Hilden, Germany) with IndiSpin® QIAcube® HT pathogen kit (INDICAL Bioscience, Leipzig, Germany). Phocine herpesvirus 1 and MS2 bacteriophage were spiked as external control at 10^6^ and 10^7^ copies to monitor extraction procedure and PCR amplification efficiency. One blank control was included in each batch of extraction and PCR detection to indicate any potential contamination (Liu et al., 2013). Nucleic acid extracts were eluted in a final volume of 100 μL and stored at −80°C.

### 2.4 Design of qPCR assays (dry lab)

Primer and probe design was performed following the published guidelines (Rodríguez et al., 2015; Behzadi and Ranjbar, 2019). *Staphylococcus* genus-specific 16S rRNA qPCR was carried out with primers (Tsingke Biological Technology) and probe (Thermo Fisher Scientific, Shanghai, China) designed for this study (Table 2). Each designed primer or probe was first BLAST searched to evaluate *in silico* specificity. None of them exhibited significant similarities to other genera (data not shown). Primers and probes of six CoNS species were designed based on the gene targets described in previous publications (Kwok et al., 1999; Hirotaki et al., 2011; Pizauro et al., 2017; Kim et al., 2021) and formulated into two panels (Table 2). Namely, panel 1 included *S. epidermidis*, *S. haemolyticus*, and *S. simulans*. Panel 2 consisted of *S. hominis*, *S. capitis*, and *S. caprae*. Designed primers and probes were BLAST searched, and none had significant similarities across *Staphylococcus* species or other genera (data not shown).

**Table 2:** PCR design used in current study.

**Multiplex qPCR assays**										
Species	Target gene	Accession number	Fragment size (bp)	Primers/Probe sequences (5'-3')	Reporter dye	Primers/Probe concn (μM)	% Efficiency	LOD*^a^* (copies/ml)	% Inclusivity	% Specificity
**Panel 1**										
*S. epidermidis*	*cydB*	CP035288 (1802782- 1802913)	132	F: CAACTGCTCTAACAATTTCAGAAGG	FAM	0.4	96.06	1260 (35.11±0.39)*^b^*	100 (7/7)*^c^*	100 (0/140)*^c^*
				R: AAAGAACTGAAACAATGGCTAAGAA		0.4				
				P: TGAGCATGGCACACATATTG		0.2				
*S. haemolyticus*	*cydB*	CP025031 (1814950- 1815091)	142	F: AGAAACCTGCACCAAAGTCAA	VIC	0.9	89.85	3000 (36.58±0.68)	100 (5/5)	100 (0/142)
				R: GCCTGCTCAAGATGATGTAGATA		0.9				
				P: AGGTCATGCTTAATGGATTACACAACT		0.25				
*S. simulans*	*hsp60*	LS483313 (1092458- 1092555)	98	F: GGACCAGGCAGAACTTTAGAT	TAMRA	0.9	105.00	400 (36.64±0.79)	100 (5/5)	100 (0/142)
				R: TGTACTTCACTTCCGGCATATT		0.9				
				P: AAAGAAGGCGACACTGTGGT		0.25				
**Panel 2**										
*S. hominis*	*nuc*	AB598389 (865-964)	100	F: GTTTAACCGTTTCTGGTGTATCAA	FAM	0.4	96.63	600 (34.91±0.56)	100 (1/1)	100 (0/146)
				R: GATCGGGTTGTAGATGGAGATAC		0.4				
				P: CGTACCTTAATTTGCTCACCATTT		0.2				
*S. capitis*	*dnaJ*	CP053957 (1293059- 1293153)	95	F: TCATTCTTCGGTGGTGGTTC	VIC	0.4	90.29	700 (36.09±0.95)	N/A*^e^*	100 (0/147)
				R: GCTTCTTCAAAGGTAACAGTCATAG		0.4				
				P: CAACGTGATCCTAATGCGCCA		0.2				
*S. caprae*	encoding gene*^d^*	CP031271 (149459- 149522)	64	F: TACATATGCGCCAGGTGAGA	TAMRA	0.4	89.35	700 (36.27±1.07)	100 (1/1)	100 (0/146)
				R: ACTGAGTTGACCTGCTTCTTTA		0.4				
				P: CAGTGAAAGCACGTGTAACAA		0.2				
***Staphylococcus* genus-specific 16S rRNA qPCR**										
	Target gene	Accession number	Fragment size (bp)	Primers/Probe sequences (5'-3')	Reporter dye	Species	% Efficiency	LOD *^f^* (copies/ml)	% Inclusivity*^g^*	% Specificity*^h^*
	16S rRNA	MF678919.1 (68-378)	311	F: TGGATAACCTACCTATAAGACTG	FAM	*S. epidermidis*	99.26	1260	100 (83/83)	100 (0/64)
				R: ATCCGAAGACCTTCATCACTC		*S. haemolyticus*	92.35	300		
				P: CGGGAAACCGGRGCTAAT		*S. simulans*	89.76	3800		
						*S. hominis*	96.09	260		

*^a^* Concentration of the lower limit of detection, LOD.

*^b^* Mean Cq value of 20 times of LOD detections ± standard deviation (SD).

*^c^* In brackets, the number of positive tests against all isolates tested for inclusivity and specificity, respectively.

*^d^* Target gene for *S. caprae* is Gram-positive signal peptide protein encoding gene.

*^e^* No *S. capiti*s strain was available for the present study.

*^f^* Mean Cq value of 20 times of LOD detections ± SD for each species: *S. epidermidis* 34.88 ± 0.57, *S. haemolyticus* 35.97 ± 0.84, *S. simulans* 34.22 ± 0.24, *S. hominis* 34.32 ± 0.38.

*^g^* Inclusivity for *Staphylococcu*s genus specific 16S rRNA qPCR.

*^h^* Specificity for *Staphylococcus* genus specific 16S rRNA qPCR.

### 2.5 qPCR setup (wet lab)

16S rRNA qPCR amplification was done in a final volume of 10 μL (AgPath-ID™ One-Step RT-PCR, Thermo Fisher Scientific): 5 μL of 2 × RT-PCR buffer, 0.4 μL of 25 × Enzyme mix, 2.0 μL of template, primers and probe at 900 nM and 250 nM final concentration, respectively. PCR conditions were set as following: an initial denaturation at 95°C for 10 min, then 40 cycles of 95°C for 15 s and 60°C for 1 min in QuantStudio 7 Flex Real-Time PCR system (Thermo Fisher Scientific, Massachusetts, United States).

CoNS qPCR conditions were optimized and each PCR reaction consisted of 5 μL of 2 × AgPath-ID™ One-Step RT-PCR buffer, 0.4 μL of 25 × Enzyme mix, 2.0 μL of template, primers and probe (Sangon Biotech, Shanghai, China) at concentrations specified in Table 2. PCR program started with an initial denaturation step at 95°C for 10 min, followed by 40 cycles of 95°C for 15 s and extension at 53°C for 1 min. All PCR results were analyzed with QuantStudio Real-Time PCR software v1.7.1. Quantification cycle (Cq) was determined by baseline threshold method.

### 2.6 Specificity and sensitivity of qPCR assays (wet lab)

Specificity of both *Staphylococcus* genus-specific 16S rRNA qPCR assay and the 3-plex qPCR panels was evaluated by testing 147 clinical isolates, including 19 CoNS strains (Supplementary Table S1). The linearity was tested with five 10-fold serial dilutions of genomic DNA or synthetic materials (10^7^ to 10^3^ copies/ml) at triplicates to construct standard curve. The lower limit of detection (LOD) was determined as the lowest concentration with 100% positive for 20 times. Cutoff value of *Staphylococcus* genus by 16S rRNA qPCR was determined to be 33 to rule out laboratory contamination. Cq values of 16S rRNA assay and the corresponding CoNS targets obtained from CoNS isolates were compared, and ΔCq was calculated to be used for determination of the primary CoNS in clinical specimens.

### 2.7 Repeatability and reproducibility of multiplex qPCR (wet lab)

The repeatability was assessed by testing 20 replicates at two (DNA extraction of four species) or three (synthesized fragments of *S. capitis* and *S. caprae*) different DNA concentrations within one experiment (Supplementary Table S2). For reproducibility, five replicates at different DNA concentrations as stated above were tested four times over two successive days, namely 20 times in total. The repeatability and reproducibility of each panel were calculated and represented by coefficient of variation (CV) (Supplementary Table S2).

### 2.8 Clinical testing with 16S rRNA qPCR and multiplex qPCR

Clinical specimens (one per patient) were first screened by *Staphylococcus* genus-specific 16S rRNA qPCR, followed by amplicon sequencing for confirmation of clinical specificity. Specimens with Cq value less than 33 were then further tested with multiplex CoNS qPCR. Then, the positive results were confirmed by re-amplifying longer amplicon for sequencing, with primers from up- and down-stream flanking sequences (130–200 bp in length, Tsingke Biological Technology) by conventional PCR (ProFlex™ PCR system, Thermo Fisher Scientific) (Supplementary Table S3). Each PCR reaction was carried out in 20 μL, containing 10 μL of 2 × Taq Plus Master Mix II (Vazyme, Nanjing, China), 2 μL of template, 0.4 μL of each primer (10 μM). The PCR reactions consisted of an initial denaturation at 95°C for 3 min, followed by 40 amplification cycles of denaturation at 95°C for 15 s, annealing at 60°C (58°C for *S. simulans*) for 30 s and extension at 72°C for 1 min, ending with a final extension at 72°C for 10 min. Amplicons were visualized with agarose gel electrophoresis and Sanger sequenced (Tsingke Biological Technology).

### 2.9 Statistical analysis

Statistical analyses were performed with SPSS software version 26. χ^2^ or Fisher’s exact tests and Student’s *t* test or Mann–Whitney *U* test were used to analyze categorical variables or continuous variables, respectively. *p*-values <0.05 were considered statistically significant.

## 3 Results

### 3.1 Characteristics of patients included in the current study

A total of 279 orthopedic patients hospitalized in a regional hospital from rural China were enrolled. Suspected infection was clinically diagnosed in 153 patients (Group A), while the remaining 126 patients showed no sign of infection (Group B). As shown in Table 1, there was no difference in age, sex, and the length of hospitalization between the two groups. The diagnosis upon admission had significant difference (*p* < 0.001) with open injury as the majority in Group A (54.25% versus 24.60% in Group B) and closed injury in Group B (69.84% versus 27.45% in Group A). Significant difference (*p* < 0.001) was observed in fracture sites with pelvic limb predominant in Group B (36.51% versus 15.69% in Group A).

### 3.2 Isolation of *Staphylococcus* spp. by culture

Of the 279 specimens, 34 *S. aureus* and 11 CoNS isolates were obtained by culture, including six *S. epidermidis*, three *S. haemolyticus*, and two *S. simulans*. A few CoNS colonies were isolated from additional six specimens but considered contamination without further speciation because of low abundance.

### 3.3 Analytical performance of qPCR assays

*Staphylococcus* genus-specific 16S rRNA qPCR was established with efficiencies between 89.76% and 99.26% for *S. epidermidis*, *S. haemolyticus*, *S. simulans*, and *S. hominis*, respectively (*R*^2^ = 0.9939, 0.9984, 0.9952, 0.9985). The inclusivity and specificity were both 100% (Table 2). Due to the ubiquitousness of *Staphylococcus* spp. and high sensitivity of qPCR assay targeting 16S rRNA, a Cq cutoff of 33 was used to preclude potential contamination.

The specificity of CoNS panels was tested with 147 clinical isolates, including 19 CoNS strains, 64 *S. aureus* strains, and 64 strains of other genera, to elucidate the cross-reaction with genetically close-related species (Supplementary Table S1). Given the fact that no *S. capitis* isolate was available for the present study, the inclusivity for other five species was 100% and the cross-reactivity was 0% for all six species. Accordingly, PCR efficiency of each species was between 89.35% and 105% with *R*^2^ of 0.9995, 0.9997, 0.9981, 1.000, 0.9970, and 0.9908, respectively. LOD was in the range of 4 × 10^2^ to 3 × 10^3^ copies/ml (Table 2). Assay repeatability ranged from 0.9% to 3.1% and reproducibility from 0.6% to 4.7% (Supplementary Table S2). Cq values of CoNS species showed good correlation with those of 16S rRNA qPCR on 17 CoNS isolates (R^2^ = 0.8926). ΔCq between the two PCR methods was calculated as mean values of Cq differences ±2 times of standard deviation (SD) (3.80 + 0.97 × 2 = 5.74). So ΔCq of 6 was used to assign the primary CoNS types in clinical samples based on the corresponding 16S rRNA qPCR results. Supplementary Figure S1 showed the representative amplification plots.

### 3.4 Clinical testing for *Staphylococcus* genus and CoNS species

For the clinical specimens, two nucleic acid extraction methods were employed, and no difference was observed (data not shown). All negative controls showed no PCR amplification. Supplementary Figure S2 summarized the overall test results. A total of 30.11% (84/279) of specimens were positive by 16S rRNA qPCR, and all confirmed by amplicon sequencing, of which 79 samples were further assayed for CoNS differentiation with five excluded due to low sample volume. CoNS qPCR panels confirmed ten culture positives, while one *S. epidermidis* culture positive sample was negative for *Staphylococcus* genus-specific 16S rRNA qPCR. Additionally, 30, 14, 13, 19, 8, and 3 samples amplified for *S. epidermidis*, *S. haemolyticus*, *S. simulans, S. hominis*, *S. capitis*, and *S. caprae*, respectively. Sanger sequencing of long amplicons flanking the qPCR targeted fragments was employed to confirm these excessive detections. 79 of the 97 positives were successfully re-amplified for longer amplicons yielding the expected sequences (Supplementary Table S4). The unconfirmed results had higher Cq values compared with the confirmed (*p <* 0.001).

Based on ΔCq addressed above, the primary *Staphylococcus* species were determined as one of the 6 CoNS interrogated for 41.77% (33/79) of the specimens. Of note, another 33 of the 79 specimens were positive for *S. aureus* by both culture and qPCR detection (unpublished data). Among the 33 CoNS specimens, 23 were identified with single CoNS species and 10 with multiple species. Worth noting is that one sample was *S. aureus* by culture but positive for both *S. epidermidis* and *S. hominis* by qPCR. Strikingly, one sample was tested as *S. capitis* by culture but mixed infection of *S. caprae* and *S. epidermidis* by qPCR, which was confirmed by whole genome sequencing (data not shown). *S. caprae* was not detected by either culture or qPCR with ΔCq criteria. Taken together, positive rate for CoNS of the 79 specimens was 12.66% (10/79) by conventional culture method but 41.77% by multiplex qPCR.

### 3.5 Clinical relevance of CoNS with orthopedic infection

**Figure 2:**
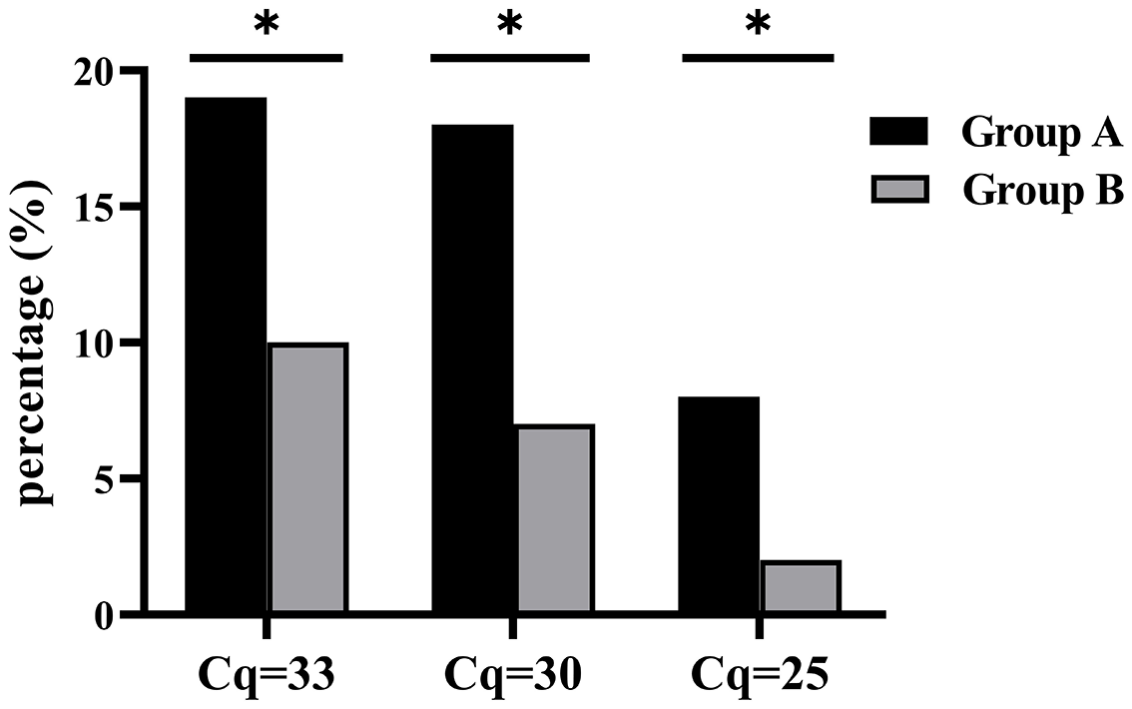
Quantity-dependent CoNS association with infection. Bar stands for the percentage of CoNS positives in either Group A or B at different Cq cutoffs. *p < 0.05.

Based on genus-specific 16S rRNA qPCR results, 43.14% (66/153) of patients in Group A were tested *Staphylococcus*-positive versus 14.29% (18/126) in Group B [odds ratio, OR, 4.55 (2.52–8.23)]. A quantitative association with infection was observed, showing increased OR with increased pathogen quantity, which was in reverse relationship with Cq values. Similar trend remained when only CoNS cases were considered, with 21 cases in Group A of 118 patients and 12 of 123 patients in Group B [OR as 2.17 (1.01–4.66) for 16S rRNA Cq cutoff of 33, 2.95 (1.23–7.06) for Cq 30, and 4.97 (1.03–23.99) for Cq 25, Figure 2; Table 3]. The sample size was relatively small for analyzing each individual species, but *χ*^2^ analysis showed the association of *S. simulans* with infection (Table 3). Additionally, the detection of other *Staphylococcus* species exhibited statistical difference between the two groups (*p* = 0.024) (Table 3).

The clinical features were further evaluated between the two groups of patients, i.e., 33 CoNS-positive and 195 negative for *Staphylococcus* genus (Table 4 and Supplementary Table S5). CoNS-positive group had longer hospital stay (median of 20 (15, 33) days versus 13 (7, 22) days, *p* = 0.003) and higher proportion of open injury (66.67%, 22/33 versus 34.36%, 67/195, *p* < 0.001) while lower proportion of closed injury (15.15%, 5/33 versus 56.41%, 110/195, *p* < 0.001).

**Table 3:** The association of CoNS with orthopedic infections.

	Group A (*n* = 118)*^a^*	Group B (*n* = 123)*^a^*	*P* value
*S. epidermidis*	11 (9.32)	10 (8.13)	0.497
*S. haemolyticus*	4 (3.39)	3 (2.44)	0.703
*S. simulans*	8 (6.78)	1 (0.81)	**0.013**
*S. hominis*	4 (3.39)	1 (0.81)	0.179
*S. capitis*	1 (0.85)	3 (2.44)	0.631
CoNS*^b^*	21 (17.80)	12 (9.76)	**0.043**
Other *Staphylococcus* species*^c^*	10 (8.47)	3 (2.44)	**0.024**

*^a^* 34 *S. aureus-*positive cases identified by qPCR were excluded from analysis, i.e. 32 in Group A and 2 in Group B. Due to low sample volume, three and one sample was further excluded from Group A or B, respectively (Supplementary Figure S2).

*^b^* All six interrogated CoNS species in this study.

*^c^* Positive for *Staphylococcus* genus-specific 16S rRNA qPCR but negative for both *S. aureus* and the six interrogated CoNS species in this study.

Statistical significance was marked in bold.

**Table 4:** Characteristics of 228 patients grouped as CoNS-positive by multiplex qPCR and staphylococci-negative by 16 s rRNA qPCR.

Characteristics	CoNS-positive (*n* = 33)	Staphylococci-negative (*n* = 195)	*p* value
Age (years)			0.819
Median	51	51	
Range	10-85	13-94	
Gender (*n*, %)			0.092
Male	27 (81.82)	131 (67.18)	
Female	6 (18.18)	64 (32.82)	
Hospitalization (days)			**0.003**
Median	20	13	
Range	4-90	1-96	
Diagnosis (*n*, %)			**<0.001**
Open injury	22 (66.67)*^a^*	67 (34.36)*^b^*	
Closed injury	5 (15.15)*^a^*	110 (56.41)*^b^*	
Postoperation	6 (18.18)	18 (9.23)	
Cause of injury (*n*, %)			**0.028**
Traffic accident	4 (12.12)	15 (7.69)	
Trauma	19 (57.58)	127 (65.13)	
Spontaneous disease	2 (6.06)	36 (18.46)	
Cutting	2 (6.06)	6 (3.08)	
Others	6 (18.18)*^a^*	11 (5.64)*^b^*	
Fracture site			0.301
NO	18 (54.55)	92 (47.18)	
Upper limb	2 (6.06)	19 (9.74)	
Pelvic limb	6 (18.18)	58 (29.74)	
Head and face	0 (0.00)	5 (2.56)	
Trunk	3 (9.09)	6 (3.08)	
Whole body	4 (12.12)	15 (7.70)	
Injured site (*n*, %)			**0.029**
Upper extremity	7 (21.21)	30 (15.39)	
Lower extremity	15 (45.46)*^a^*	128 (65.64)*^b^*	
Trunk	4 (12.12)*^a^*	4 (2.05)*^b^*	
Whole body	7 (21.21)	32 (16.41)	
Head and face	0 (0.00)	1 (0.51)	
Laboratory findings (*n*, %)*^c, d^*			*OR* (95% *CI*)
White blood cell count, WBC			
Normal	25 (78.13)	101 (76.52)	
High	7 (21.87)	26 (19.70)	1.088 (0.424-2.791)
Low	0 (0.00)	5 (3.78)	-*^e^*
Lymphocyte%, LYMPH%			
Normal	15 (46.88)	68 (51.52)	
High	2 (6.24)	5 (3.79)	1.813 (0.321-10.254)
Low	15 (46.88)	59 (44.69)	1.153 (0.520-2.555)
Lymphocyte, LYMPH			
Normal	30 (93.74)	125 (94.70)	
High	1 (3.13)	1 (0.76)	4.167 (0.253-68.540)
Low	1 (3.13)	6 (4.54)	0.694 (0.081-5.987)
Percentage of neutrophils, NEU%			
Normal	23 (71.88)	94 (71.21)	
High	8 (25.00)	35 (26.52)	0.934 (0.382-2.282)
Low	1 (3.12)	3 (2.27)	1.362 (0.135-13.706)
Neutrophils, NEU			
Normal	23 (71.88)	96 (72.73)	
High	9 (28.12)	32 (24.24)	1.174 (0.493-2.797)
Low	0 (0.00)	4 (3.03)	-*^e^*
C-reactive protein, CRP			
Normal	4 (50.00)	18 (33.96)	
High	4 (50.00)	35 (66.04)	0.500 (0.111-2.248)
Erythrocyte sedimentation rate, ESR			
Normal	2 (28.57)	9 (25.71)	
High	5 (71.43)	26 (74.29)	0.900 (0.148-5.489)
Procalcitonin, PCT			
Normal	2 (40.00)	9 (36.00)	
High	3 (60.00)	16 (64.00)	0.844 (0.118-6.031)
D-dimer, DD			
Normal	9 (81.82)	68 (80.95)	
High	2 (18.18)	16 (19.05)	0.944 (0.186-4.801)

^*a*, *b*^ Differences between groups with statistical significance.

*^c^* Not all enrolled patients were tested for listed biomarkers and only available data were included for analysis.

*^d^* Reference range of listed biomarkers: WBC 4.0-10×10^9^ /L, LYMPH% 20-40%, LYMPH 0.8-4.0×10^9^ /L, NEU% 46-76.5%, NEU 2-7×10^9^ /L, CRP 0-8 mg/L, ESR male 0-15 and female 0-20 mm/h, PCT 0-0.05 ng/ml, DD 0-1 μg/ml.

*^e^* Data not available.

Statistical significance was marked in bold.

## 4 Discussion

CoNS have been gradually recognized as an important cause of nosocomial infections and indwelling foreign body-related infections (Huebner and Goldmann, 1999; von Eiff et al., 2002; Becker et al., 2014; Heilmann et al., 2019). Considering their concerning degree of capabilities for antibiotic resistance and biofilm formation, valid and precise species-level identification is critical for clinical diagnosis and antibiotic regimens. For orthopedic infections, failure to identify the etiology may result in missing the window for appropriate treatment, which may lead to chronic infection, amputation, or even patient death. According to the China Antimicrobial Surveillance Network (CHINET), CoNS isolates are generally considered contaminants or colonizers on skin face; therefore, only colonies from blood and sterile fluid specimens have been included and analyzed (Hu et al., 2018, 2022). Reports of CoNS from China tend to focus on a single specific species—mostly *S. epidermidis—*of a single syndrome—mostly bloodstream infection in a relatively limited geographic region (Guo et al., 2019; Cui et al., 2022; Li et al., 2023). Therefore, to the best of our knowledge, the well-timed and comprehensive surveillance of CoNS across China remains rare, and even more scarce for orthopedic infections.

Nowadays, PCR, especially qPCR reinforced by fluorescent probes, has become a powerful molecular approach for pathogen identification in both clinical research and diagnosis. Previous studies established PCR methods for CoNS detection (Hirotaki et al., 2011; Kim et al., 2021), which were performed on food samples or clinical isolates (Campos-Peña et al., 2014; Kim et al., 2018). The current study combined genus-specific 16S rRNA qPCR to first screen for *Staphylococcus*-positive specimens with two sets of 3-plex qPCR assays to simultaneously identify six clinically relevant CoNS. A Cq-dependent algorithm was established to identify the primary CoNS in *Staphylococcus*-positive specimens. The positive rate ranged from 5.06% (*S. capitis*) to 26.58% (*S. epidermidis*) by qPCR assays while between 2.53% (*S. simulans*) and 6.33% (*S. epidermidis*) by the routine culture method, which did not detect any *S. hominis*, *S. capitis*, or *S. caprae*. Considering that PCR is widely acknowledged to be less affected by prior antibiotic treatment and has even succeeded in detecting bacterial persisters 1 month post antimicrobial therapy in some cases and acknowledging the power of polymicrobial detection by multiplex qPCR panels, the reported qPCR methods had considerable advantage in CoNS infection investigation (Canvin et al., 1997; van der Heijden et al., 1999; Higgins et al., 2022). To be aware, when none of the 6 CoNS species was detected with ΔCq ≤ 6 of 16S rRNA, it might indicate the existence of other *Staphylococcus* species that were not included in the present assays. For instance, one specimen was *S. epidermidis* positive with a Cq value of 31.91 by multiplex qPCR, but the Cq of genus-specific 16S rRNA qPCR was 21.43, coinciding with the fact *S. aureus* was isolated by culture and detected by qPCR at Cq 23.77.

Worth mentioning, one specimen, identified as *S. capitis* by culture but mixed *S. epidermidis* and *S. caprae* by qPCR and WGS, confirmed the previous report that VITEK® 2 system could not distinguish *S. caprae* from *S. capitis* well (d’Ersu et al., 2016). From a genetics perspective, *S. capitis* and *S. caprae* are closely related and prone to be misclassified, which conceivably impedes their identification and discrimination in clinical practice (Sun et al., 2020; Heath et al., 2023). Put together, the presented multiplex qPCR assays demonstrated adequate analytical specificity and appreciable power of discrimination between genetically closely related species.

In the current study, CoNS were detected more frequently in patients with clinically suspected infection. CoNS colonize on skin surface (Becker et al., 2014; Severn and Horswill, 2023); therefore, CoNS isolates tend to be ignored in clinical practice, particularly when the number of colonies is low. For example, in the current study, CoNS colonies were isolated from six specimens but discarded as contamination by clinical laboratory, while qPCR detected two as *S. simulans*, one as *S. epidermidis*, one as other *Staphylococcus* species, and two at low level of 16S rRNA amplification above cutoff. Thus, through accurate quantification, qPCR enabled more precise analysis to rule out the interference of contamination from skin flora and to extrapolate the relevance of CoNS with infection, which turned stronger at higher bacterial loads with OR increased from 2.17 (1.01–4.66) for Cq cutoff of 33 to 4.97 (1.03–23.99) for Cq 25. This evidently demonstrated a quantitative association of CoNS with orthopedic infection. Continuous surveillance may help refine this clinical cutoff and characterize the relevance of CoNS with orthopedic infection at individual species level.

Statistical analysis between Groups A and B elucidated that open injury and trauma were significantly related to suspected infections. Risk factors of CoNS-associated infections were consistent with previous studies, such as male sex. In addition, open injury and lower extremity were also proved to be characteristics of CoNS infections (Kortram et al., 2017). To be aware, the enrolled patients were mainly farmers or migrant workers performing heavy physical labor on daily base. Many of them would not seek medical care unless conditions seriously deteriorated. Longer hospital stay observed with CoNS-positive patients may be due to the delayed hospitalization to certain extent. Other than that, CoNS are empirically neglected in clinical practice, which would result in delayed diagnosis and inadequate treatment, being costly for both patients and healthcare system (Becker et al., 2014; Severn and Horswill, 2023). Unfortunately, the available data on commonly accepted inflammatory biomarkers, such as CRP and ESR, were not sufficient for statistical review. Further investigation is required to elucidate the association between biomarkers and CoNS infections observed in this study (Taccone et al., 2009; Zheng et al., 2017; Ishibuchi et al., 2021).

The current study has a few limitations. Firstly, swabs have been argued over lower efficiency and less sensitivity compared with tissue samples. Considerable discordance was also observed between superficial and deep swabs (Higgins et al., 2022). This study followed the standard procedure established in a local hospital from rural China, where tissue, blood clots, or syringe aspirate were occasionally available and could be evaluated for comparison. Secondly, only six CoNS species were included in qPCR panels. However, all of them have clinical significance according to not only the literature but more importantly also the historical results from the clinical laboratory of the participating hospital. Other clinical associated CoNS, such as *S. lugdunensis* and *S. saprophyticus,* were not included, because they were rarely reported in China, neither by our hospital (Liu C. et al., 2012; Qian et al., 2021). About one-fifth of the *Staphylococcus* detection belonged to other species, surprisingly showing the relevance with infection, and thereby requiring further characterization and potential speciation panel to be developed. Small sample size was another study limitation, making it infeasible to analyze the pathogen specific association with infection and to determine the quantitative relationship. Furthermore, the positive detection of *S. capitis* and *S. caprae* was relatively low in this study, which can alternatively be explained by the fact *S. capitis* and *S. caprae* are relatively less encountered in human-, animal-, or food-derived samples.

In summary, we have established a rapid and accurate qPCR method, including *Staphylococcus* genus-specific 16 s rRNA qPCR, and species-specific multiplex qPCR, to precisely identify six CoNS species with clinical relevance. CoNS were found to be associated with orthopedic infections in a quantitative manner. This could potentially facilitate the elucidation of etiological landscape of CoNS in orthopedic infections and the improvement of treatment decision-making in the real world.

## Data availability statement

The original contributions presented in the study are publicly available. This data can be found here: https://www.ncbi.nlm.nih.gov/bioproject/PRJNA1080311.

## Ethics statement

The studies involving humans were approved by ethics boards of both participant institutes: Qingdao University, Qingdao, China and Qingdao Huangdao Traditional Chinese Medicine Hospital, Qingdao, Shandong, China. The studies were conducted in accordance with the local legislation and institutional requirements. Written informed consent for participation in this study was provided by the participants’ legal guardians/next of kin.

## Author contributions

YingW: Conceptualization, Data curation, Formal analysis, Investigation, Methodology, Project administration, Resources, Supervision, Validation, Visualization, Writing – original draft, Writing – review & editing. CL: Data curation, Formal analysis, Investigation, Methodology, Software, Validation, Visualization, Writing – review & editing. WX: Conceptualization, Data curation, Investigation, Project administration, Resources, Writing – review & editing. YC: Conceptualization, Data curation, Investigation, Project administration, Resources, Writing – review & editing. LY: Data curation, Investigation, Software, Validation, Writing – review & editing. DZ: Data curation, Investigation, Software, Validation, Writing – review & editing. XG: Investigation, Methodology, Project administration, Resources, Writing – review & editing. YingdW: Data curation, Investigation, Software, Validation, Writing – review & editing. YaW: Data curation, Investigation, Software, Validation, Writing – review & editing. YL: Methodology, Project administration, Software, Supervision, Writing – review & editing. JH: Investigation, Methodology, Project administration, Resources, Supervision, Writing – review & editing. JL: Data curation, Formal analysis, Funding acquisition, Investigation, Methodology, Project administration, Resources, Supervision, Validation, Writing – original draft, Writing – review & editing.
