# Supplementary material for: Association of coagulase-negative staphylococci with orthopedic infections detected by in-house multiplex real-time PCR
Source: Front Microbiol. 2024 Jun 7;15:1400096. doi: 10.3389/fmicb.2024.1400096 (PMC11193334; doi:10.3389/fmicb.2024.1400096)
Supplement: Supplementary file 1 [file Table_1.docx]

Supplementary Material

Association of Coagulase-negative Staphylococci with Orthopedic Infections Detected by in House Multiplex Real Time PCR

Ying Wang^1†^, Chao Liu^1†^, Wenbo Xia^2^, Yanxiang Cui^3^, Linhong Yu^4^, Dan Zhao^1^, Xiaoxuan Guan^3^, Yingdi Wang^1^, Yani Wang^1^, Yisong Li^1^, Jianqiang Hu^2^*, Jie Liu^1^*

*** Correspondence:** Jie Liu, jl5yj@qdu.edu.cn; Jianqiang Hu, 13853239702@163.com

# Supplementary Tables

**TABLE S1.** Clinical isolates used for PCR validation and results of both *Staphylococcus* genus specific 16S rRNA qPCR and multiplex qPCR assays.

| Species | No. of isolates (n=147) | qPCR target (number of qPCR-positive isolates) | | | | | | |
| --- | --- | --- | --- | --- | --- | --- | --- | --- |
|  |  | 16S rRNA | *S. epidermidis* | *S. haemolyticus* | *S. simulans* | *S. hominis* | *S. capitis* | *S. caprae* |
| ***Staphylococcus* spp.** | **83** |  |  |  |  |  |  |  |
| *S. aureus* | 64 | 64 | 0 | 0 | 0 | 0 | 0 | 0 |
| Coagulase-negative staphylococci (CoNS) | **19** | **19** | **7** | **5** | **5** | **1** | **0** | **1** |
| *S. epidermidis* | 7 | 7 | 7 | 0 | 0 | 0 | 0 | 0 |
| *S. haemolyticus* | 5 | 5 | 0 | 5 | 0 | 0 | 0 | 0 |
| *S. simulans* | 5 | 5 | 0 | 0 | 5 | 0 | 0 | 0 |
| *S. hominis* | 1 | 1 | 0 | 0 | 0 | 1 | 0 | 0 |
| *S. capitis* | N/A*^a^* | 0*^b^* | 0 | 0 | 0 | 0 | 0 | 0 |
| *S. caprae* | 1 | 1 | 0 | 0 | 0 | 0 | 0 | 1 |
| ***Other genera*** | **64** | **0** | **0** | **0** | **0** | **0** | **0** | **0** |
| *Acinetobacter baumannii* | 3 | 0 | 0 | 0 | 0 | 0 | 0 | 0 |
| *Acinetobacter pittii* | 2 | 0 | 0 | 0 | 0 | 0 | 0 | 0 |
| *Acinetobacter soli* | 1 | 0 | 0 | 0 | 0 | 0 | 0 | 0 |
| *Aeromonas hydrophila* | 2 | 0 | 0 | 0 | 0 | 0 | 0 | 0 |
| *Enterobacter aerogenes* | 1 | 0 | 0 | 0 | 0 | 0 | 0 | 0 |
| *Enterobacter cloacae* | 8 | 0 | 0 | 0 | 0 | 0 | 0 | 0 |
| *Escherichia coli* | 14 | 0 | 0 | 0 | 0 | 0 | 0 | 0 |
| *Klebsiella pneumoniae* | 10 | 0 | 0 | 0 | 0 | 0 | 0 | 0 |
| *Enterococcus faecalis* | 2 | 0 | 0 | 0 | 0 | 0 | 0 | 0 |
| *Proteus mirabilis* | 4 | 0 | 0 | 0 | 0 | 0 | 0 | 0 |
| *Proteus penneri* | 1 | 0 | 0 | 0 | 0 | 0 | 0 | 0 |
| *Proteus vulgaris* | 1 | 0 | 0 | 0 | 0 | 0 | 0 | 0 |
| *Serratia marcescens* | 2 | 0 | 0 | 0 | 0 | 0 | 0 | 0 |
| *Streptococcus agalactiae* | 2 | 0 | 0 | 0 | 0 | 0 | 0 | 0 |
| *Branhamella catarrhalis* | 1 | 0 | 0 | 0 | 0 | 0 | 0 | 0 |
| *Providencia rettgeri* | 1 | 0 | 0 | 0 | 0 | 0 | 0 | 0 |
| *Pseudomonas aeruginosa* | 7 | 0 | 0 | 0 | 0 | 0 | 0 | 0 |
| *Pseudomonas stutzeri* | 2 | 0 | 0 | 0 | 0 | 0 | 0 | 0 |

*^a^* No *S. capitis* strain was available for the present study.

*^b^* 0 stands for negative PCR results because of no *S. capitis* strain available.

**TABLE S2.** Repeatability and reproducibility testing of multiplex qPCR panel assays.

|  | log_10_ (copies/ml) | Repeatability (CV, %) | Reproducibility (CV, %) |
| --- | --- | --- | --- |
| **Panel 1** |  |  |  |
| *S. epidermidis* | 7 | 1.5 | 2.4 |
|  | 5 | 1.4 | 3.3 |
| *S. haemolyticus* | 6 | 1.1 | 0.8 |
|  | 4 | 1.9 | 1.7 |
| *S. simulans* | 7 | 0.9 | 4.6 |
|  | 5 | 2.2 | 4.7 |
| **Panel 2** |  |  |  |
| *S. hominis* | 6 | 1.0 | 0.6 |
|  | 4 | 1.0 | 1.1 |
| *S. capitis* | 6 | 2.7 | 2.5 |
|  | 4 | 2.4 | 2.1 |
|  | 3 | 2.6 | 2.6 |
| *S. caprae* | 6 | 3.1 | 3.0 |
|  | 4 | 2.6 | 2.4 |
|  | 3 | 2.9 | 2.6 |

TABLE S3. Conventional PCR with longer amplicon.

| Species | Target gene | Accession number | Fragment size (bp) | Primers/Probe sequences (5'-3') |
| --- | --- | --- | --- | --- |
| **Panel 1** | | | | |
| *S. epidermidi*s | *cydB* | CP035288 | 132 | F: CAACTGCTCTAACAATTTCAGAAGG |
|  |  |  |  | R: AAAGAACTGAAACAATGGCTAAGAA |
| *S. haemolyticus* | *cydB* | CP025031 | 142 | F: AGAAACCTGCACCAAAGTCAA |
|  |  |  |  | R: GCCTGCTCAAGATGATGTAGATA |
| *S. simulans* | *hsp60* | LS483313 | 188 | F: GGACCAGGCAGAACTTTAGAT |
|  |  |  |  | R: GCCTCCTGTTATTTGTTATGATTG |
| **Panel 2** | | | | |
| *S. hominis* | *nuc* | AB598389 | 184 | F: GTTTAACCGTTTCTGGTGTATCAA |
|  |  |  |  | R: ACAGGGCCATTTAAAGACGA |
| *S. capitis* | *dnaJ* | CP053957 | 130 | F: GGTTTAGCGGAGGTGGATTT |
|  |  |  |  | R: GCTTCTTCAAAGGTAACAGTCATAG |
| *S. caprae* | encoding  gene*^a^* | CP031271 | 144 | F: TACATATGCGCCAGGTGAGA |
|  |  |  |  | R: TGCATATTTTCAGGCTTTGC |

*^a^* Target gene for *S. caprae* is Gram-positive signal peptide protein encoding gene.

**TABLE S4.** Comparison between multiplex qPCR assays and Sanger sequencing.

| Species | qPCR positive | Sequencing positive | Cq value (median, range) | Sequencing negative | Cq value (median, range) | *P* value |
| --- | --- | --- | --- | --- | --- | --- |
| **Panel 1** |  |  |  |  |  |  |
| *S. epidermidis* | 35 | 33 | 34.09 (30.50-36.33) | 2 | 39.22*^a^* (38.47, 39.96) | 0.039 |
| *S. haemolyticus* | 17 | 13 | 35.74 (32.58-38.49) | 4 | 39.24 (36.40-39.64) | 0.089 |
| *S. simulans* | 15 | 11 | 31.14 (27.28-34.03) | 4 | 37.00 (33.79-38.58) | 0.013 |
| **Panel 2** |  |  |  |  |  |  |
| *S. hominis* | 19 | 14 | 34.44 (32.12-37.02) | 5 | 36.50 (36.15-37.78) | 0.016 |
| *S. capitis* | 8 | 6 | 31.94 (28.36-36.84) | 2 | 37.59*^a^* (36.63, 38.54) | 0.096 |
| *S. caprae* | 3 | 2 | 34.90*^a^* (33.88, 35.91) | 1 | 37.17*^b^* | 0.221 |
| **Sum** | **97** | **79** | **33.46 (31.11-36.49)** | **18** | **37.78 (36.29-38.73)** | **<0.001** |

*^a^* Average Cq value of two available samples.

*^b^* Cq value of the only one sample.

**TABLE S5.** Laboratory findings of 228 patients grouped as CoNS-positive by multiplex qPCR and staphylococci-negative by 16s rRNA qPCR.

| Biomarkers*^a,b^* | CoNS-positive (n=33) | Staphylococci-negative (n=195) | *OR* (95% *CI*) |
| --- | --- | --- | --- |
| Red blood cell count, RBC | | |  |
| Normal | 28 (87.50) | 111 (84.09) |  |
| High | 2 (6.25) | 1 (0.76) | 7.929 (0.694-90.600) |
| Low | 2 (6.25) | 20 (15.15) | 0.396 (0.087-1.797) |
| Hematocrit, HCT | | |  |
| Normal | 27 (84.38) | 90 (68.18) |  |
| High | 1 (3.12) | 2 (1.52) | 1.667 (0.145-19.096) |
| Low | 4 (12.50) | 40 (30.30) | 0.333 (0.109-1.016) |
| Hemoglobin, HGB | | |  |
| Normal | 26 (81.24) | 100 (75.76) |  |
| High | 3 (9.38) | 5 (3.79) | 2.308 (0.518-10.290) |
| Low | 3 (9.38) | 27 (20.45) | 0.427 (0.120-1.519) |
| Albumin, ALB | | |  |
| Normal | 20 (68.97) | 74 (69.81) |  |
| High | 2 (6.90) | 0 (0.00) | -*^c^* |
| Low | 7 (24.13) | 32 (30.19) | 0.809 (0.311-2.104) |
| α1-globulin, α1-QDB | | |  |
| Normal | 23 (79.31) | 61 (57.55) |  |
| High | 6 (20.69) | 44 (41.51) | **0.362 (0.136-0.962)** |
| Low | 0 (0.00) | 1 (0.94) | -*^c^* |
| β-globulin, β-QDB | | |  |
| Normal | 25 (86.21) | 91 (85.85) |  |
| High | 1 (3.45) | 14 (13.21) | 0.260 (0.033-2.074) |
| Low | 3 (10.34) | 1 (0.94) | **10.920 (1.088-109.578)** |
| γ-Globulin, GAMMA | | |  |
| Normal | 22 (75.86) | 73 (68.87) |  |
| High | 7 (24.14) | 32 (30.19) | 0.726 (0.282-1.870) |
| Low | 0 (0.00) | 1 (0.94) | -*^c^* |
| Total bilirubin, TBIL | | |  |
| Normal | 22 (75.86) | 91 (85.85) |  |
| High | 7 (24.14) | 15 (14.15) | 1.930 (0.702-5.304) |
| Indirect bilirubin, IBIL | | |  |
| Normal | 23 (79.31) | 95 (88.79) |  |
| High | 6 (20.69) | 12 (11.21) | 2.065 (0.701-6.085) |
| Alkaline Phosphatase, ALP | | |  |
| Normal | 25 (86.21) | 99 (92.52) |  |
| High | 4 (13.79) | 8 (7.48) | 1.980 (0.552-7.106) |
| Creatine Kinase, CK | | |  |
| Normal | 17 (60.71) | 67 (63.21) |  |
| High | 7 (25.00) | 34 (32.08) | 0.811 (0.307-2.145) |
| Low | 4 (14.29) | 5 (4.71) | 3.153 (0.763-13.024) |
| Sialic acid, SA | | |  |
| Normal | 20 (71.43) | 63 (58.88) |  |
| High | 6 (21.43) | 44 (41.12) | 0.430 (0.160-1.156) |
| Low | 2 (7.14) | 0 (0.00) | -*^c^* |

*^a^* Not all patients were tested for listed biomarkers and only available data were included for analysis.

*^b^* Reference range of listed biomarkers: RBC 3.5-5.5×10^12^ /L, HCT 35-50%, HGB 110-160 g/L, ALB 60.00-71.00%, α1-QDB 1.40-2.90%, β-QDB 8.00-13.00%, GAMMA 8.00-16.00%, TBIL 0-21 μmol/L, IBIL 0-17 μmol/L, ALP 30-120 U/L, CK 38-174 U/L, SA 400-700 mg/L.

*^c^* Data not available.

Statistical significance was marked in bold.


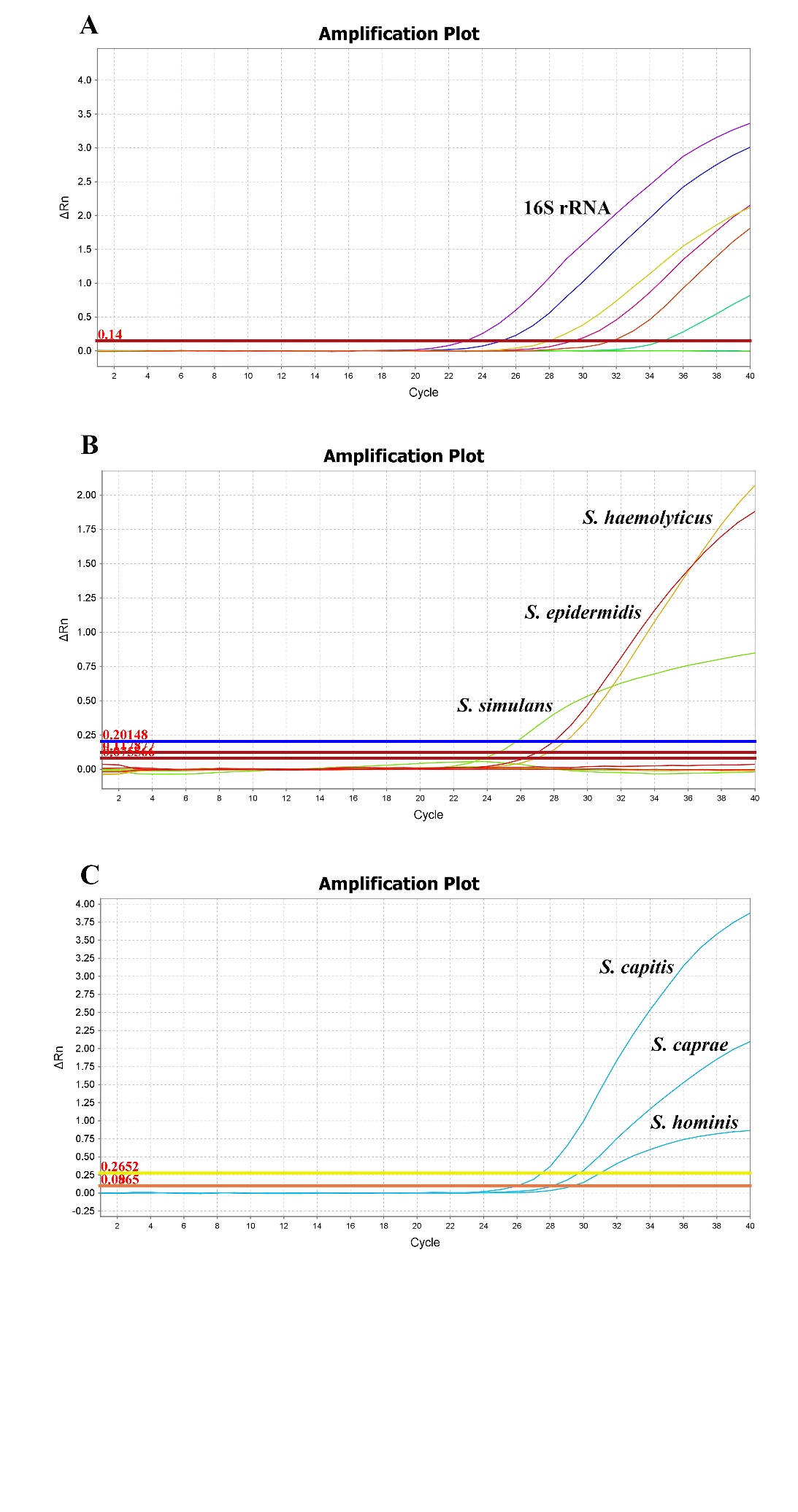


**Figure S1.** Amplification plots of *Staphylococcus* genus-specific 16S rRNA qPCR (A), multiplex qPCR of CoNS detection Panel 1 (B, including *S. epidermidis*, *S. haemolyticus*, and *S. simulans*), and Panel 2 (C, including *S. hominis*, *S. capitis*, and *S. caprae*).

**
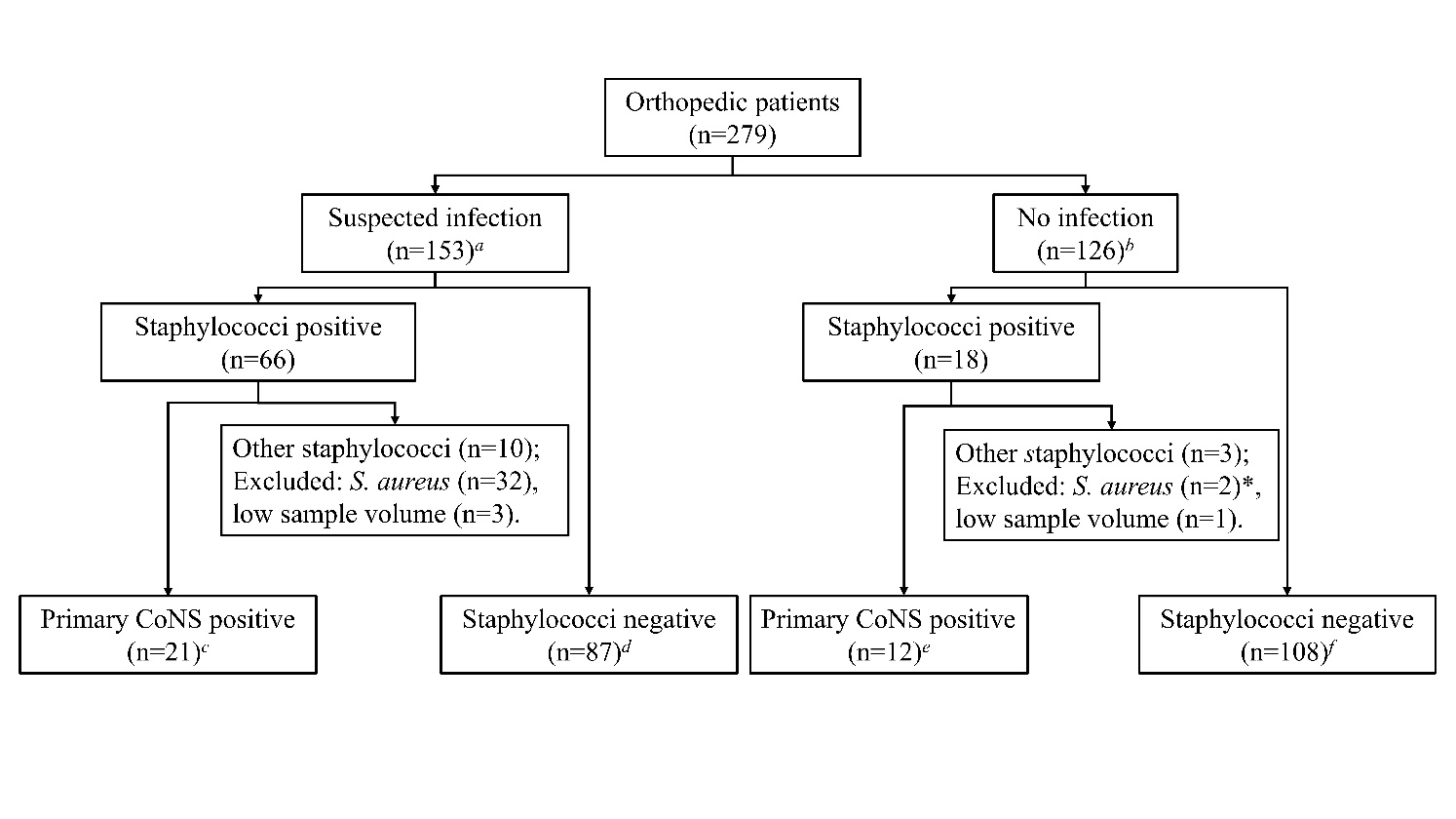
**

**Figure S2.** Summary of the test results of qPCR on clinical specimens. Further analysis was performed, including demographic difference between Suspected infection (a) and No infection (b) as shown in Table 1, primary CoNS association with infection (c/d versus e/f) (Table 1, Figure 2), and clinical difference between primary CoNS positive (c and e) and staphylococci negative (d and f) patients (Table 3 and Supplementary Table S5). *One *S. aureus* positive sample was not tested with CoNS panels in Result 3.4 due to low sample volume.
